# Cloning and functional characterization of seed-specific *LEC1A* promoter from peanut (*Arachis hypogaea* L.)

**DOI:** 10.1371/journal.pone.0242949

**Published:** 2021-03-22

**Authors:** Guiying Tang, Pingli Xu, Pengxiang Li, Jieqiong Zhu, Guangxia Chen, Lei Shan, Shubo Wan

**Affiliations:** 1 Bio-Tech Research Center, Shandong Academy of Agricultural Sciences / Shandong Provincial Key Laboratory of Crop Genetic Improvement, Ecology and Physiology, Jinan, Shandong, China; 2 College of Life Science, Shandong Normal University, Jinan, Shandong, China; 3 Shandong Academy of Grape, Jinan, Shandong, China; National Taiwan University, TAIWAN

## Abstract

LEAFY COTYLEDON1 (LEC1) is a HAP3 subunit of CCAAT-binding transcription factor, which controls several aspects of embryo and postembryo development, including embryo morphogenesis, storage reserve accumulation and skotomorphogenesis. Herein, using the method of chromosomal walking, a 2707bp upstream sequence from the ATG initiation codon site of *AhLEC1A* which is a homolog of Arabidopsis *LEC1* was isolated in peanut. Its transcriptional start site confirmed by 5’ RACE was located at 82 nt from 5’ upstream of ATG. The bioinformatics analysis revealed that there existed many tissue-specific elements and light responsive motifs in its promoter. To identify the functional region of the *AhLEC1A* promoter, seven plant expression vectors expressing the *GUS* (β-glucuronidase) gene, driven by 5’ terminal series deleted fragments of *AhLEC1A* promoter, were constructed and transformed into Arabidopsis. Results of GUS histochemical staining showed that the regulatory region containing 82bp of 5’ UTR and 2228bp promoter could facilitate GUS to express preferentially in the embryos at different development periods of Arabidopsis. Taken together, it was inferred that the expression of *AhLEC1A* during seed development of peanut might be controlled positively by several seed-specific regulatory elements, as well as negatively by some other regulatory elements inhibiting its expression in other organs. Moreover, the *GUS* expression pattern of transgenic seedlings in darkness and in light was relevant to the light-responsive elements scattered in *AhLEC1A* promoter segment, implying that these light-responsive elements harbored in the *AhLEC1A* promoter regulate skotomorphogenesis of peanut seeds, and *AhLEC1A* expression was inhibited after the germinated seedlings were transferred from darkness to light.

## Introduction

Seed development is a complex procedure of the flowering plant in life cycle, which can conceptually be divided into two distinct phases: embryo morphogenesis and seed maturation [1, 2]. Lots of genes highly and specifically expressed in different developmental processes highlight the importance of transcriptional regulations for proper seed formation [3]. The Arabidopsis *LAFL* genes coding for LEAFY COTYLEDON1 (LEC1), ABSCISIC ACID INSENSITIVE3 (ABI3), FUSCA3 (FUS3), and LEC2, respectively form a network involved in regulating seeds development [4–7].

LEC1 is a central regulator controlling embryogenesis and seed maturation in *Arabidopsis thaliana* [8–10]. It expresses primarily in the embryo and endosperm, particularly during seed development [9, 11]. Loss of *LEC1* function causes a pleiotropic phenotype, including cotyledon with trichomes, distortions in suspensor-cell specification, defects in storage protein and lipid accumulation, embryo desiccation-intolerance in developing seeds, and leaf primordia initiation [8, 12, 13]. The expression of many genes involved with maturation processes are downregulated in *lec1* mutant seeds [9, 14, 15]. Moreover, the role of *LEC1* was also demonstrated by analyzing its gain-of-function mutant. For example, overexpression of *LEC1* in developing seeds elevates the contents of seed storage macromolecule, as well, upregulates the key genes involved in storage protein and lipid accumulation in a number of plant species [16–20]. Genome-wide analysis of *LEC1* occupancy and interactome indicated that LEC1 regulate many genes involved in embryo development [14, 15]. Recently, several researches indicated that LEC1, a central regulator of seed development, interacts with different combinations of ABI3, bZIP67, FUS3, and other TFs to regulate diverse developmental processes at different stages of seed development [21, 22].

LEC1 also participates in regulating post-embryonic growth during the developmental transition from germination seeds to seedlings. In higher plants, rapid elongation of hypocotyl in germinating seeds can be induced by darkness. However, *lec1* mutant causes a reduced capability for hypocotyl elongation and apical hook formation [14, 23]. Additionally, the expression of *LEC1* was also detectable in etiolated seedlings [24, 25], and the phenotype of longer hypocotyls and higher expression levels of the genes involved in etiolated growth were observed in LEC1-overexpression plants [26]. A *LEC1* gain-of-function mutant turnip (*tnp*), displayed the partial de-etiolation at dark-grown condition, including inhibited hypocotyls elongation, and activated SAM (shoots apical meristem) [27].

Despite of such comprehensive knowledge about *LEC1* in the model plant *Arabidopsis*, much less is known about the expression patterns and functions in other higher plants. Here, the 2707bp 5’ flanking region of peanut *AhLEC1A* was isolated, and the GUS expression profiles driven by a series of deletion in its 5’ flanking region were characterized in transgenic Arabidopsis. The results showed that the regulatory region containing 82bp of 5’ UTR and 2228bp promoter could specifically regulate *AhLEC1A* expressing in developing seeds. Thus, the *AhLEC1A* promoter could be utilized as a seed-preferential promoter for plant genetic engineering.

## Materials and methods

### Plant materials and growth conditions

Peanut (*Arachis hypogaea* L. cv. Luhua 14) seeds, *Arabidopsis thaliana* L. (Ecotypes Col) seeds, *Escherichia* coli strain DH5α, pCAMBIA3301 plasmid and *Agrobacterium tumefaciens* strain GV3101 used in the present study were maintained at our laboratory. Peanut plants were grown in the experimental field of Shandong Academy of Agricultural Sciences. Roots, stems and leaves of 14-day seedlings, flowers, and the developing seeds were collected and kept in -80°C refrigerator for isolation of total RNA.

### Cloning of the 5’ flanking region of *AhLEC1A*

The peanut genomic DNA was isolated from Luhua 14 leaves using CTAB method [28]. Genome walking was performed to isolate the 5’ flanking regulatory region. According to the BD Genome Walker Universal Kit (Clontech, USA) manufacturer’s instructions, each of 2.5 μg genomic DNA was digested with four restriction enzyme *DraI*, *EcoRV*, *PvuII*, and *StuI* respectively; and then the digested samples were connected with the BD Genome-Walker adaptor resulting in the library containing digestions by *DraI*, *EcoRV*, *PvuII*, and *StuI* (LD, LE, LP, and LS). Based on the sequence of *AhLEC1A* genomic DNA, two nested gene-specific primers (GSP), LEC1AGSP1-2 and LEC1AGSP2-2, were designed. The first round of PCR reaction was done in a 25μL reaction system using an AP1 provided by Kit and LEC1A GSP1-2 as 5’ terminal and 3’ terminus primer, and 1μL DNA of each library as template. The nested PCR reaction was also performed using the same volume and conditions with primers AP2 and LEC1AGSP2-2, and 1μL of the 10-fold diluted primary PCR products as template. The specific PCR fragments from the second round reaction were isolated and inserted into the vector pEASY-T3. The recombinants harboring the target gene were validated by two-way sequencing using ABI3730 model DNA sequencer. The primer and adaptor sequences of this assay were listed in Table 1.

**Table 1:** List of primers used in the study.

Serial No.	Name	Sequence (from 5’ to 3’)	Feature
1	BD GenomeWalker adaptor	GTAATACGACTCACTATAGGGCACGCGTGGTCGACGGCCCGGGCTGGT	No.1-5 used for the amplification of 5’ flanking sequence of *AhLEC1A*
2	LEC1AGSP1-2	TCAACCCAGAGGTAGTGGTAGGAAGG	
3	LEC1AGSP2-2	TGATAACCGTGAAAGCCTCCTCCAGT	
4	AP1	GTAATACGACTCACTATAGGGC	
5	AP2	ACTATAGGGCACGCGTGGT	
6	GeneRacer RNA Oligo	CGACUGGAGCACGAGGACACUGACAUGGACUGAAGGAGUAGAAA	No.6-10 used for the localization of the transcriptional start site of *AhLEC1A* gene
7	TSS LECGSP1-1	TCTTTTGCGTCGTCGGAGATTTTAGC	
8	TSS LECGSP2-2	TGATAACCGTGAAAGCCTCCTCCAGT	
9	5’ GeneRacer Primer	CGACTGGAGCACGAGGACACTGA	
10	5’ Nested Primer	GGACACTGACATGGACTGAAGGAGTA	
11	P1	CCATGGGTGTGAAGAAAGATGCAGTG	No.11-18 used for the amplification of different length promoter fragments
12	P2	AAGCTTCATTAGGGTCAAAAGAGTG	
13	P3	AAGCTTTCTTGGCAATAAATGTTGG	
14	P4	AAGCTTCCCGTTAAAAAAAATAATAAG	
15	P5	AAGCTTGTAATTTTTGGATAGCTTG	
16	P6	AAGCTTTACATGGCACGCCTCATATC	
17	P7	AAGCTTAGATCGAAACTAATTTAAG	
18	P8	AAGCTTAAAAAGTTGAACATTTTATATAG	
19	AhACTIN7-F	ATGTATGTAGCCATCCAAG	No.19-22 used for qRT- PCR analysis of *AhLEC1A*
20	AhACTIN7-R	ACCAGAGTCCAGAACAATA	
21	AhLEC1A-F	ATACTCATACAGATGATAAC	
22	AhLEC1A-R	TGTGGAACAAAAGCAGAAGT	

### Precise identification of transcription start site in *AhLEC1A*

The transcription start site of *AhLEC1A* gene was identified by 5’ RACE (rapid amplification of cDNA ends) using a 5’ RACE kit (Invitrogen GeneRacer^™^ Kit) following the instructions provided by the manufacturer. Total RNA was extracted from the developing seeds of peanut Luhua 14 using the improved CTAB method [29]. The ds-cDNA was synthesized using the full-length mRNA with RNA Oligo as template. The ds-cDNA was cloned into vector pCR4-TOPO to establish the full-length cDNA library. According to the cDNA sequence of *AhLEC1A*, two 3’ terminus gene-specific primers TSS LEC1AGSP1-1 and TSS LEC1AGSP2-2 were designed, for use in the nested PCR reaction. The 5’ terminus general primer for two rounds of PCR were GeneRacer^™^ Primer and 5’ Nested Primer. 1μL of the full-length cDNA library as got previously and a 50-fold dilution of the primary PCR product was used respectively as the template of the two rounds of PCR. The nested PCR products were collected and sequenced by ABI3730 model DNA sequencer. The primer sequences used in the assay were listed in Table 1.

### Expression analysis of *AhLEC1A* gene in various organs

The expression analysis was performed by qRT-PCR using ABI 7500 instrument. Gene-specific primers were designed according to *AhLEC1A* cDNA sequence (Table 1). The first-stand cDNAs of *AhLEC1A* were amplified using SYBR premix Ex Taq polymerase (Takara). Its relative expression level was analyzed using *AhACTIN7* as the reference gene by the 2^-ΔΔCT^ method [30]. Three sample repetitions with technical triplicates were set in the experiment.

### In-silico analysis of the *AhLEC1A* promoter for cis-regulatory elements

The cis-elements of the 5’ flanking region of *AhLEC1A* gene were analyzed using PLACE (http://www.dna.affrc.go.jp/PLACE) and Plant Cis-Acting Regulatory Elements (Plant CARE) (http://bioinformatics.psb.ugent.be/webtools/plantcare/html/).

### Plasmid construction and Arabidopsis transformation

A series of 5’ -truncated promoter sequences were obtained by PCR using a single reverse primer localized in 5’ UTR of *AhLEC1A*, and different forward primers situated in the different sites of the *AhLEC1A* promoter (The primer sequences were listed in Table 1). To construct the vector, the appropriate restriction sites were introduced into the PCR-amplified promoter (*HindIII* at the 5’ end; *NcoI* at the 3’ end). The PCR-amplified promoter was then inserted into *HindIII*/*NcoI*-digested pCAMBIA3301, replacing the cauliflower mosaic virus (CaMV) 35S promoter, producing seven deletion constructs containing various fragments (-2228 ~ +82, Q7; -1254 ~ +82, Q6; -935 ~ +82, Q5; -721 ~ +82, Q4; -617 ~ +82, Q3; -354 ~ +82, Q2; -105 ~ +82, Q1).

The constructs including Q1-Q7 and the control pCAMBIA3301 was introduced into *Agrobacterium tumefaciens* strain GV3101 using a freeze-thaw method. Transgenic Arabidopsis plants were generated by the floral dip method. The seeds of the T_0_-T_2_ generations were germinated on 1/2MS_0_ agar medium containing 10μg/L Basta. The copy number in transgenic plants was determined by segregation ratio of the plants with and without basta-resistance. The T_1_ transgenic lines with single copy gene have the 3:1 ratio of resistant plants to non-resistant plants. The homozygous lines of T_2_ generation were screened on basta-resistant 1/2MS_0_ medium. More than eight homozygous lines respectively carrying single copy gene of Q1-Q7 and the control pCAMBIA3301 were obtained. The identified transgenic plants were transferred to soil under 120 μmol·m^-2^·s^-1^light in a growth room at a temperature between 22°C and 25°C. All Arabidopsis plants grew under a 16h light/8h dark photoperiod, and 65% relative humidity.

### Histochemical GUS staining

The GUS assay was performed as described by Jefferson [31]. For each *AhLEC1A* promoter-GUS construct, at least thirty plants of T_2_ generation lines in five transgenic events were used for GUS histochemical staining. The roots and leaves at the 4-leaf stage, stems at the bolting stage, flowers, immature embryos of 6–10 days after pollination and 3-5day etiolated and de-etiolated seedlings in transgenic T_2_ lines were incubated in GUS assay buffer with 50mM sodium phosphate(7.0), 0.5mM K_3_Fe(CN)_6_, 0.5mM K4Fe(CN)_6_·3H_2_O, 0.5% Triton X-100, and 1mM X-Gluc at 37°C overnight and then cleared with 70% ethanol. The samples were examined by stereomicroscopy.

## Results

### Isolation of the promoter of *AhLEC1A* and localization of TSS

**Fig 1:**
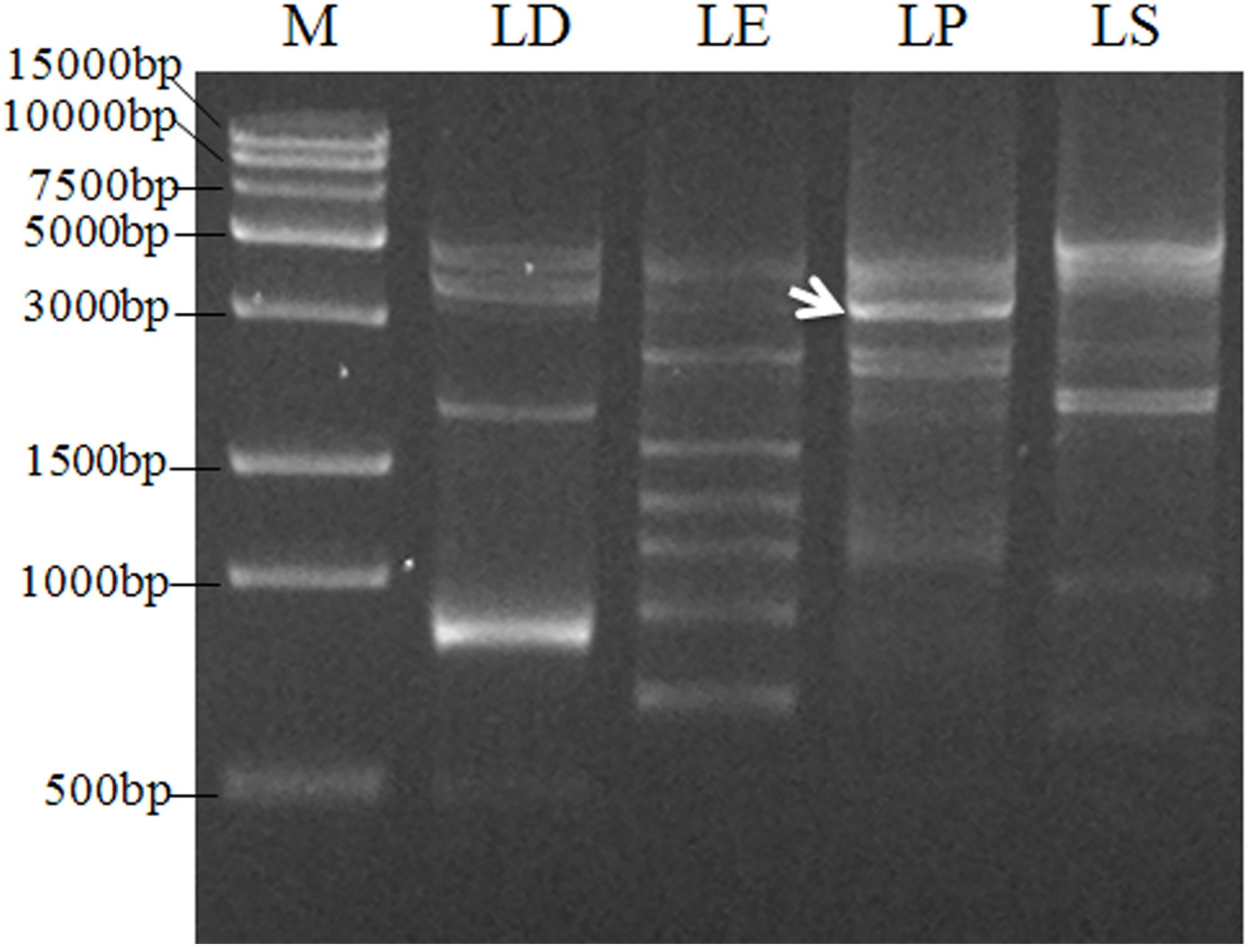
PCR amplification of 5’ flanking regulation regions of peanut *AhLEC1A* gene by chromosome walking. LD, LE, LP and LS represents the second amplification with different primary product as template respectively. The arrow indicates the targeted band for further cloning and sequencing.

**Fig 2:**
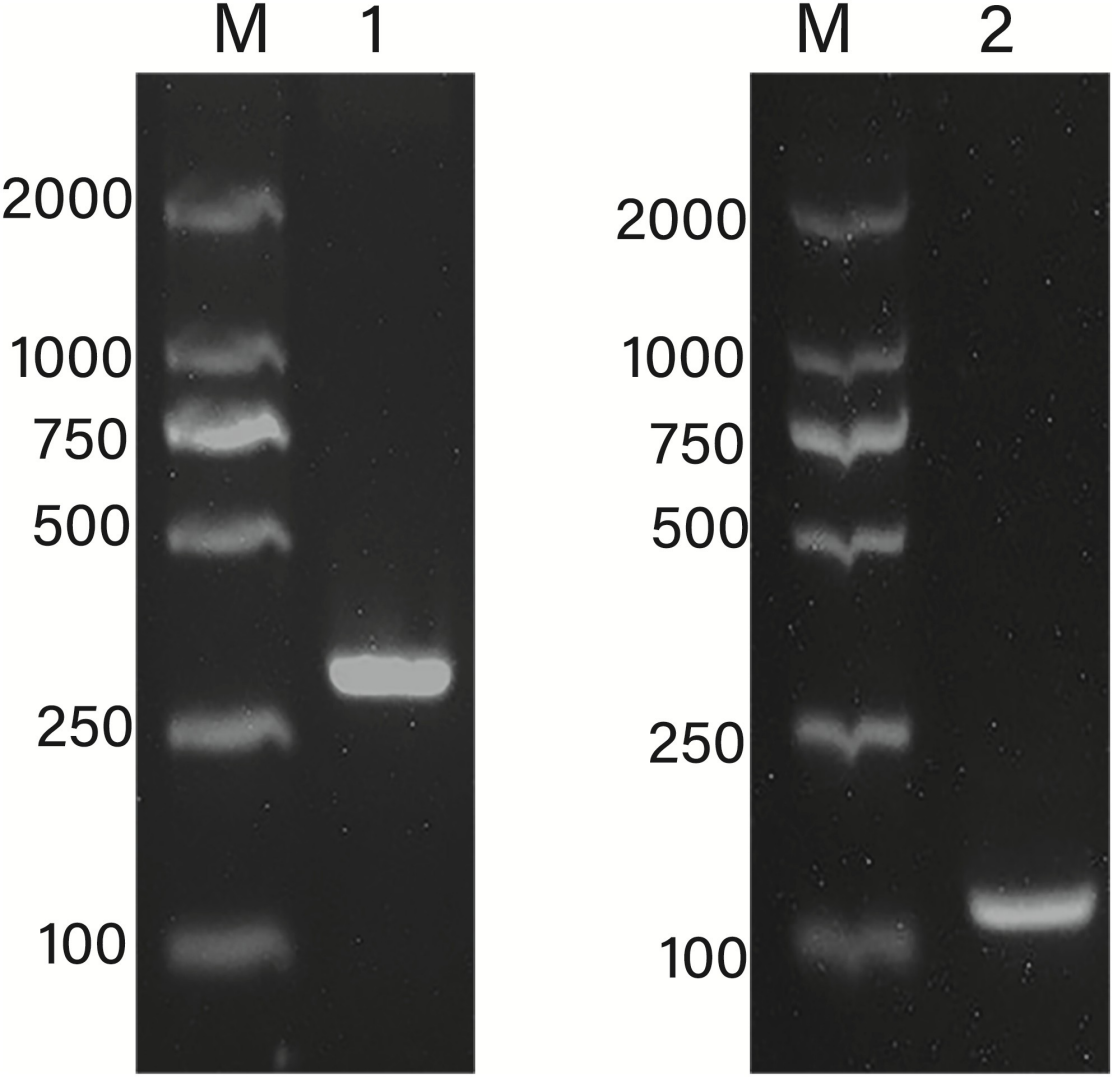
Localization of transcription start site of peanut *AhLEC1A* gene using 5’ RACE. 1-The products of the first round PCR; 2-The products of the second round PCR.

The 2739bp DNA fragment was amplified by two rounds of PCR using the method of genome walking. Its sequence analysis found that this fragment includes 2707bp of 5’ flanking region upstream from ATG and 32bp of coding sequence (Fig 1). In order to determine the transcription start site (TSS) of *AhLEC1A* gene, the nested 5’ RACE was performed to amplify the 5’-end of its cDNA. The 140bp of cDNA fragment, including the 58bp of coding region started from ATG and 82bp 5’ UTR, was isolated (Fig 2). Compared with the gDNA sequence of *AhLEC1A*, the sequence of 82bp 5’ UTR was identical to the 5’ upstream sequence of its gDNA, suggesting that the “A” located at the 82th nucleotid (nt) upstream from ATG is the TSS of *AhLEC1A* gene.

### Analysis of cis-regulatory elements in *AhLEC1A* promoter sequence

**Fig 3:**
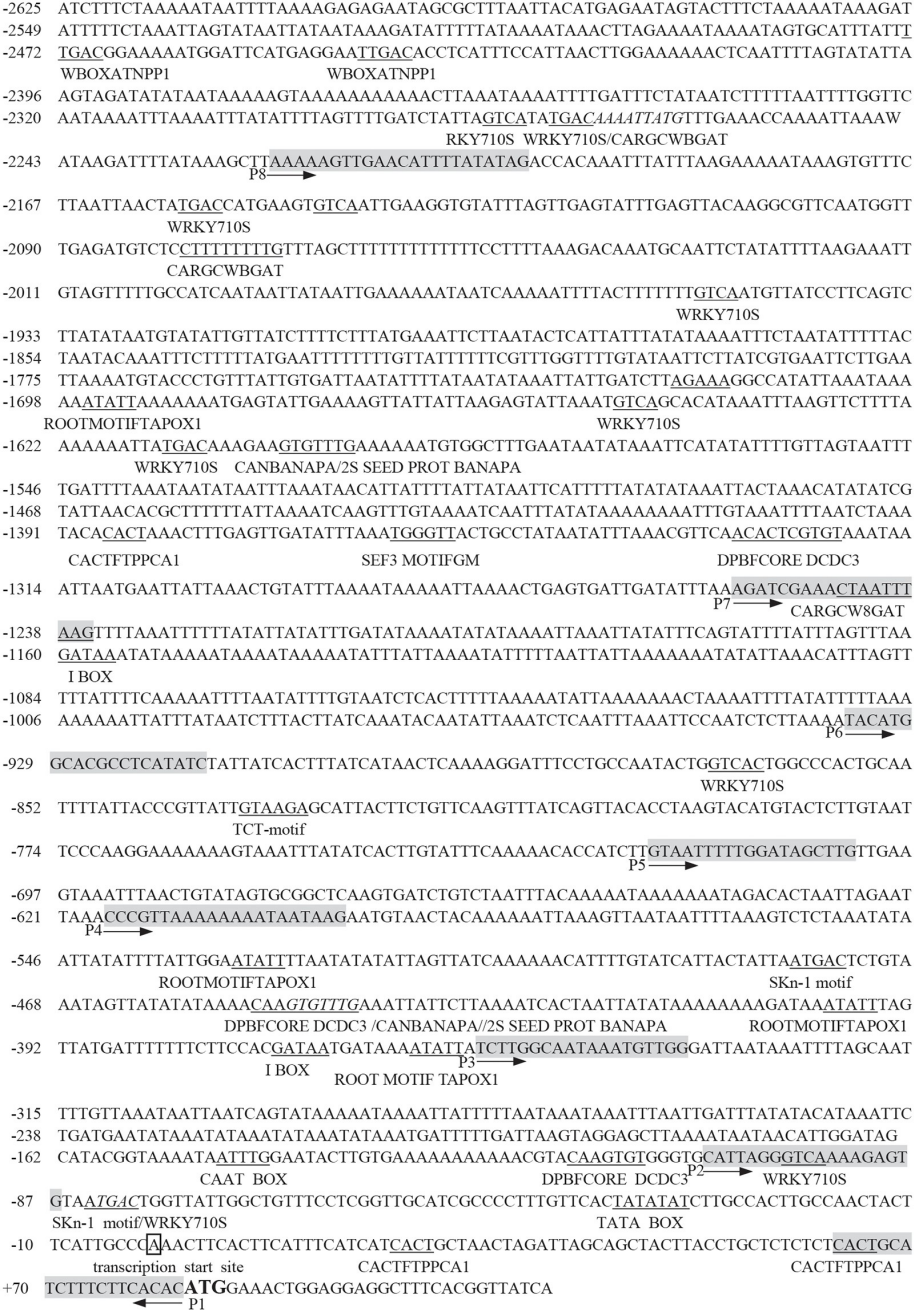
The sequence of 5’ flanking regulation region of peanut *AhLEC1A* gene and some major elements harbored in this region. The letter “A” in box represents its transcription start site (TSS), the putative regulatory elements are highlighted by underling or italicizing, and the sequences of primers P1-P8 are shading.

*In silico* analysis of 2707bp 5’ flanking region revealed that a number of putative cis-elements were present in the 2625bp of promoter region and 82bp of 5’ UTR (Fig 3). In detail, the basic promoter elements, TATA box (TATATAT) and CAAT box (CAAAT)), respectively placed at -36 ~ -30 nt and -143 ~ -148 nt. Many crucial elements required for embryo- or endosperm-specific expression and seed storage compounds accumulation scattered over the promoter, including two SKn-1 motifs (GTCAT) at -84 ~ -80 nt and -475 ~ -479 nt, two CANBANAPA elements (CNAACAC) at -442 ~ -448 nt and -1597 ~ -1603 nt, and three binding sites for AGL15 (CWWWWWWWWG) at -1245 ~ -1236 nt, -2079 ~ -2070 nt and -2272 ~ -2263 nt. In addition, four DPBFCOREDCDC3 elements (ACACNNG), previously considered to involve in embryo-specific expression and also to respond to ABA were found at -111 ~ -117 nt, -445 ~ -451 nt, -1321 ~ -1327 nt and -1330 ~ -1324 nt. We have also detected many other regulatory elements for the accumulation of seed storage compounds and embryogenesis. For instance, eight EBOX BNNAPA (CANNTG), two 2S SEED PROT BANAPA (CAAACAC) and one SEF3 MOTIF GM (AACCCA). Besides, there were some elements associated with regulating in vegetative organ development on the promoter, such as mesophyll-specific element CACTFTPPCA1 (YACT), root-specific element ROOTMOTIFTAPOX1 (ATATT) and so on. There also exist some elements involved in light responsiveness including more than a dozen I BOX (GATAA) at -366 ~ -362 nt, -372 ~ -368 nt, -405 ~ -401 nt, -357 ~ -353 nt, -511 ~ -507 nt, -810 ~ -806 nt, -905 ~ -901 nt, -913 ~ -909 nt, -983 ~ -979 nt, -1793 ~ -1789 nt, -1916 ~ -1912 nt, and -1946 ~ -1942 nt, three -10PEHVPSBD (TATTCT) at -437 ~ -432 nt, -2584 ~ -2579 nt, and -2606 ~ -2601 nt, and one TCT-motif (TCTTAC) at -830 ~ -835 nt, etc., and some other regulatory elements controlling the chloroplast genes expression, like one GT1 MOTIF PSRBCS (KWGTGRWAAWRW) at -137 ~ -126 nt, and two etiolation-induced expression elements ACGT ATERD1 (ACGT) at -122 ~ -119 nt, and -1334 ~ -1337 nt. We also identified thirteen negative regulatory elements in the promoter, among which five WBOXATNPR1 elements located in -1255 ~ -2228 nt region (-1613 ~ -1616 nt, -1649 ~ -1652 nt, -1953 ~ -1956 nt, -2144 ~ -2147 nt, and -2156 ~ -2159 nt), and four WRKY71OS elements densely distributed between the region of -2228 to -2625 nt.

### Functional analysis of the regulatory regions of the *AhLEC1A* promoter

**Fig 4:**
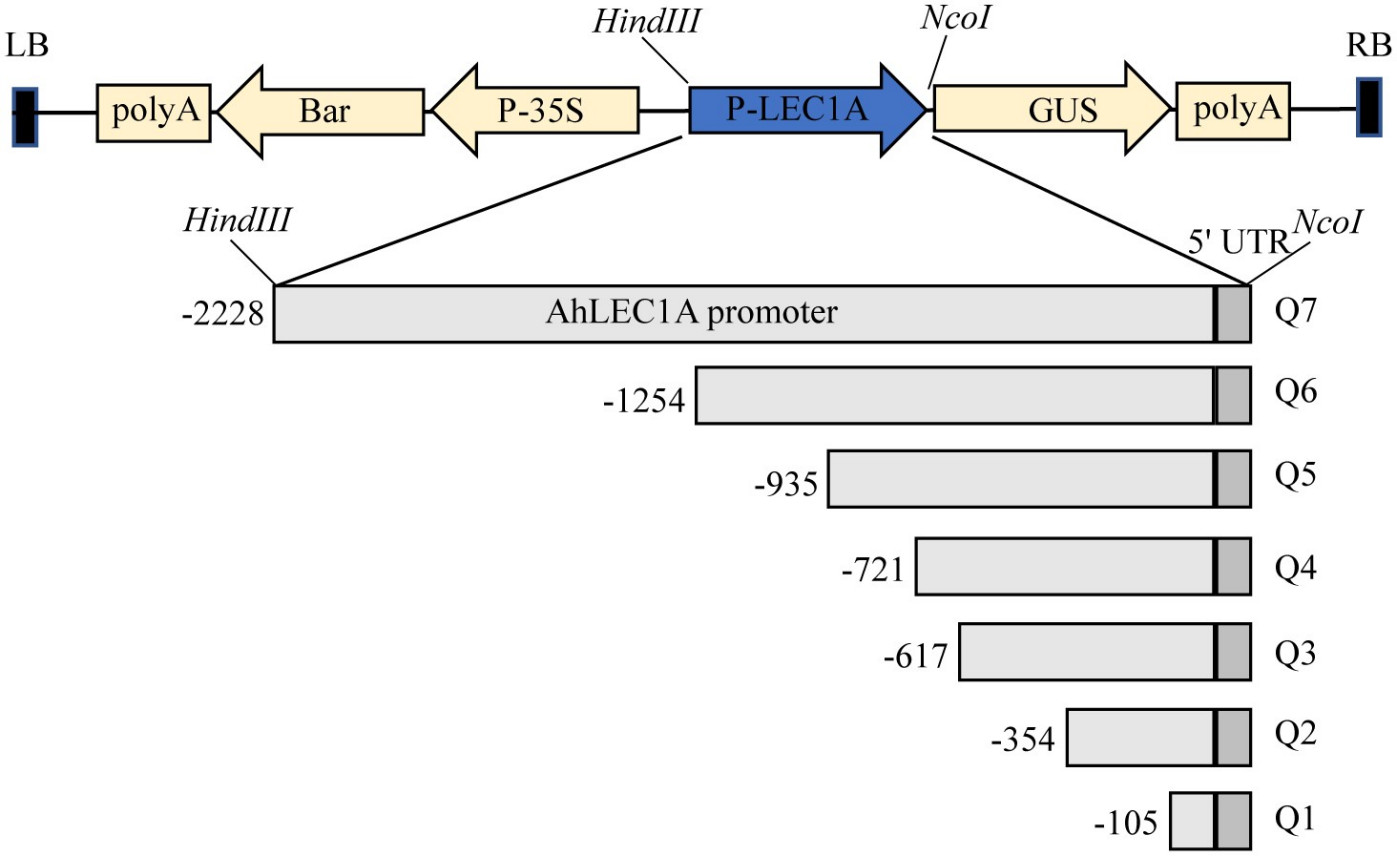
The vector diagram expressing GUS in plants driven by different length *AhLEC1A* promoters with 5’ terminal deletion. Q1~Q7 indicates its promoters with different length. The rectangles in light and dark gray respectively show the promoter region upstream of the TSS, and the 5’ UTR region of gene.

**Fig 5:**
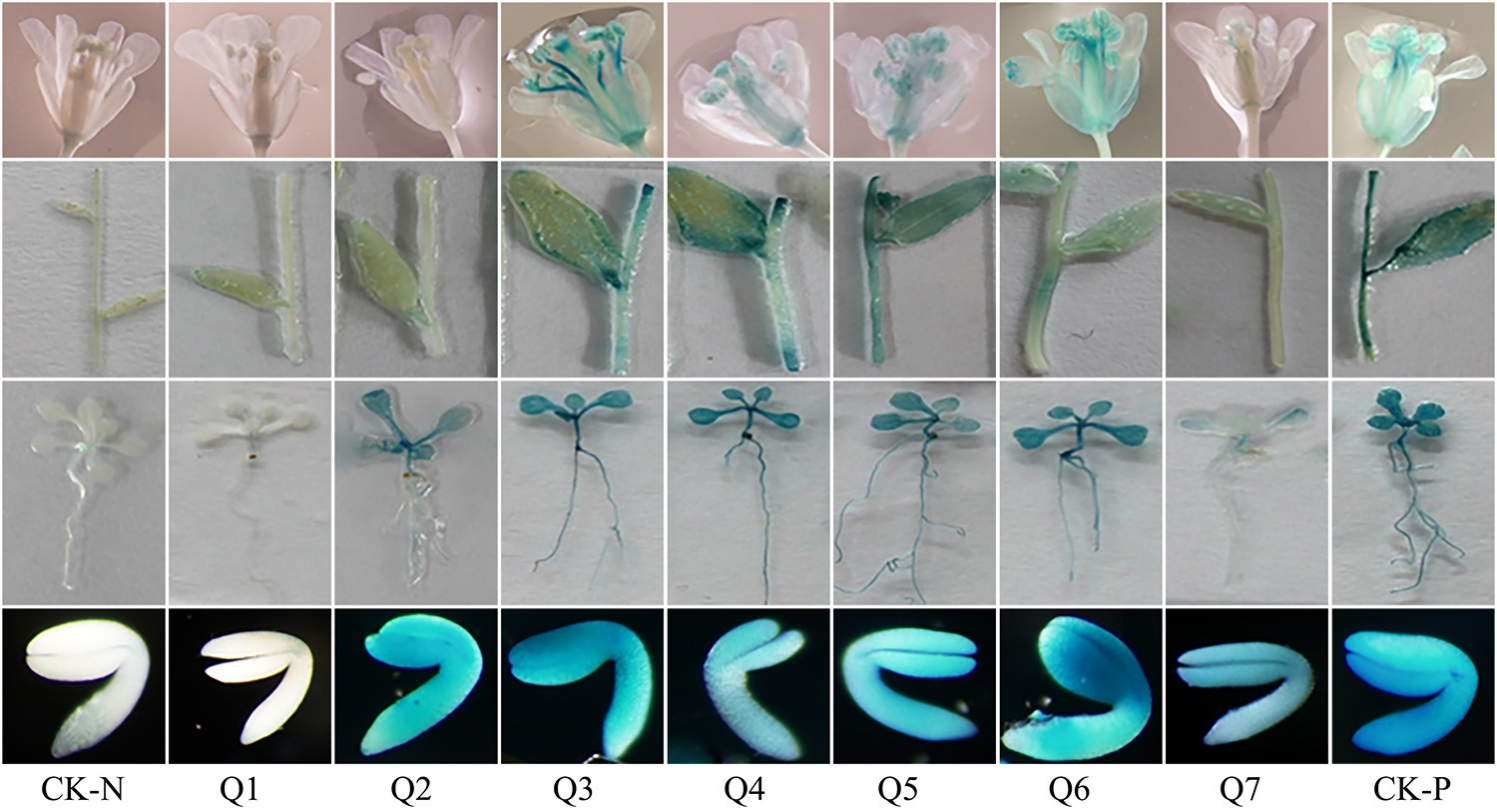
GUS Histochemical staining of different organs in transgenic Arabidopsis. Q1~Q7 respectively shows the GUS expression patterns in flower, stem and cauline leaf, and rosette leaf and root harboring different GUS expression structures, and the CK-N and CK-P respectively show the GUS expression profiles in COL, and in positive control harboring 35S::GUS constructs.

To validate the role of the crucial regulatory region in *AhLEC1A* promoter, a series of GUS expression vectors (Q1~ Q7) (Fig 4), driven by different length of the promoters with truncated 5’ terminal were established, and the GUS expression patterns in stable transgenic plants of *Arabidopsis* was investigated. In the histochemical assay, GUS expression was visualized specifically in the developing embryos of transgenic plants containing Q7 construct (including 2228bp promoter region and 82bp 5’ UTR, Fig 5). The result of *AhLEC1A* expression analysis by qRT-PCR also showed that its transcripts were higher in seeds, but lower or rarely in roots, stems, leaves and flowers (S1 Fig) Otherwise, the GUS staining was observed in all detected organs of transgenic plants carrying Q3, Q4, Q5, and Q6 construct (Fig 5). These four promoter segments are respectively 617bp, 721bp, 935bp and 1254bp in size with 5’ terminal deletion of 1611bp ~ 974bp. It was suggested that there exist some key motifs in the promoter region between -2228bp and -1255bp, which related to inhibit the expression in the other organs except for the developing seeds. Moreover, the further deletional promoter fragment Q2 with 354bp drove the GUS to express only in embryos and rosette leaves. The shortest fragment Q1 containing 105bp promoter region and 82bp 5’ UTR couldn’t drive the GUS to express in any detected organs of transgenic *Arabidopsis* (Fig 5), implying that it might be caused by the deletion of the necessary component for gene expression.

**Fig 6:**
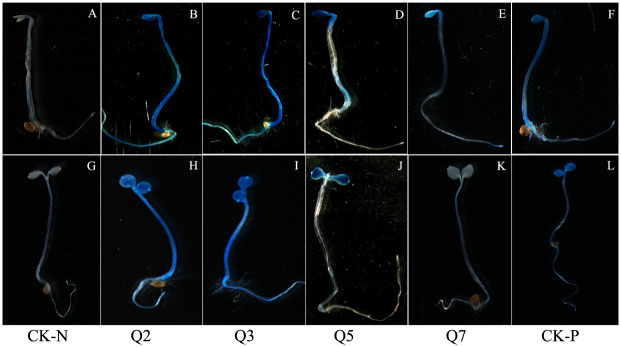
GUS staining of the seedlings germinated for 3~5d in transgenic Arabidopsis. Q2, Q3, Q5 and Q7 indicate respectively the GUS expression patterns in transgenic Arabidopsis lines harboring different GUS expression structures. CK-N and CK-P indicate the GUS expression profiles in COL and in positive control harboring 35S::GUS construct, respectively. A-F indicate the seedlings germinated for 3d in dark, and G-L represent the seedlings after transferring from dark to light for 2 days.

To explore the role of *AhLEC1A* on seedling establishment, the transgenic lines with Q7, Q5, Q3 and Q2 constructs were chosen for further analysis. Transgenic Arabidopsis seeds were kept in the dark till their germinating. The results of GUS staining indicated that the unexpanded cotyledon and apex hook of the seedlings harboring Q7 or Q5 construct showed dark blue color, and the hypocotyls were light blue. However, after the etiolated transgenic seedlings had been moved to the light for 2 days, the plants with Q7 construct hardly got dyed, and only the expanded cotyledons with Q5 construct were stained blue. By contrast, the whole seedlings with Q3 or Q2 construct were dyed dark blue under both growth conditions (Fig 6). The results suggested that there existed some negatively regulatory elements at the region of -2228bp ~ -618bp in *AhLEC1A* promoter to control the expression of *AhLEC1A* in hypocotyls and radicles at the stage of seedling formation, and some of them mentioned above might associate with light response.

## Discussion

Identifying and characterizing the 5’ flanking region of gene is helpful for revealing its temporal and spatial expression pattern, and facilitating its utilization in plant genetic engineering [32]. In the present study, we have cloned and analyzed the 5’ flanking regulatory sequence of *AhLEC1A*. Several cis-elements in *AhLEC1A* promoter, such as SKn-1, CANBANAPA (CA)_n_, AGL15, DOF core, SEF3 motif and the like, which previously were demonstrated to be required for seed development and storage accumulation, were identified. Skn-1 motif and (CA)_n_ element were reported to play a vital role in determining the seed-specific expression; the deletion of Skn-1 motif or (CA)_n_ element in *glutelin* and *napin* promoter decreased their transcription in seeds [33, 34]. The element of DOF core was considered to confer the endosperm-specific expression in *Zea mays* [35, 36]. SEF3 motif is the binding site of Soybean Embryo Factor 3, which regulate the transcription of the β-conglycinin (a storage protein) gene and participate in seed development [37, 38]. Our results of GUS staining assay revealed *GUS* gene, driven by the longest *AhLEC1A* promoter (Q7), specially expressed in the embryos of the transgenic Arabidopsis, which is well in agreement with our results of gene expression analyzed by qRT-PCR method (S1 Fig). These data showed that AhLEC1A functioned in a seed-specific manner. Otherwise, the transgenic lines with 1611bp ~ 974bp deletion constructs from 5’ terminal of Q7 promoter showed the constitutive expression at higher GUS levels in roots, rosettes, stems, flowers, and seeds. Meanwhile, in silico analysis of *AhLEC1A* promoter displayed several tissue-specific elements like mesophyll-specific element CACTFTPPCA1, root-specific element ROOTMOTIFTAPOX1 and pollen-specific element POLLEN1LELAT52 distributed on its upstream regulatory region, as well as many negatively regulatory elements including four WRKY71OS and five WBOXATNPR1 dispersed intensively in the fragment of -1225 ~ -2228bp which was deleted in Q3 ~ Q6. These results demonstrate that *AhLEC1A* expression in seed-specific pattern might be attributed to be negatively regulated its transcription in vegetative organs by some cis-element existed in the distal region of its promoter, and simultaneously to be controlled its expression in seeds positively by some seed-specific elements in the proximal region of its promoter. This regulatory model was also found in *AtLEC1* promoter of Arabidopsis [27], *D540* promoter of rice [39], and *C-hordein* promoter of barley [40].

Beyond embryogenesis and embryo development, LEC1 also regulate skotomorphogenesis of seedlings at the post-germination stage. The unexpanded cotyledons and apical hook of seedlings with Q7 construct germinated in dark dyed obviously in blue, while the whole seedlings were scarely stained after transferring to light for 2 days. It was suggested that light might repress the expression of *AhLEC1A* by recruiting some proteins to bind the particular elements in its promoter. Our results found that total 23 light-responsive elements I BOX CORE/GATA BOX (GATA) scattered on Q7 segment of *AhLEC1A* promoter, 9, 15 and 21 out of them were respectively deleted in Q5, Q3 and Q2 promoter, resulting in that in GUS assay, the staining patterns of Q2 and Q3 transgenic plants in darkness were similar to those in light, and the hypocotyls of Q5 transgenic plants were dyed in blue when growing in darkness while there were no dyeing after transferring them to light. The core sequence of I BOX, and the GATA BOX with similar function had been shown to be essential for light-regulated transcriptional activation [41–43]. Furthermore, it has been demonstrated that I BOX as a negative cis-element can inhibit the expression of *GalUR* in strawberry, and the inhibited role is strictly depended on light [44]. Yamagata et al. also found that I BOX, as a negative regulatory element, was necessary for down-regulating the expression of *cucumisin* gene by binding fruit nuclear protein in Musc melons (*Cucumis melo* L.) [45]. Previous study found that *AtLEC1* promoter exists several I BOX CORE elements, and deleting some of them localized on 5’ upstream segment from -436 nt in mutant *tnp* restrains the hypocotyl elongation of etiolated seedlings in darkness [27]. These data suggested that some of I BOX elements function as a negative regulator in response to illumination. In our GUS histochemical assay, the degree and range of dyeing changed with the number of I BOX, demonstrating that some of them might be involved in negative regulating the expression of *AhLEC1A* gene during the procedure of seedling growth from dark condition to light condition.

The *cis*- elements comparison in the promoters of *AhLEC1A* and *AhLEC1B* showed that lots of similar elements are dispersed in the both promoters, but their amounts and positions were much different (Table 2). *AhLEC1A* promoter contained a number of distinct seed-development related components such as 2S SEED PROT BANAPA, SP8BFIBSP8BIB, CANBNNAPA. However, *AhLEC1B* promoter contained numerous specific elements involved in abiotic stress or hormones responding, including GCCCORE, ASF1MOTIFCAWV, and several regulatory elements known to modulate gene expression at higher transcription level in different plant species [46]. The similarities and differences between two *AhLEC1* promoters implied that their functions might be partially same and redundant, and to some extent *AhLEC1A* and *AhLEC1B* might play different roles during the particular growth and development period of peanuts, respectively. The point of view was consistent with the study of predecessors who thought *LEC1* genes originated from a common ancestor and neofunctionalization and /or subfunctionalization processes were responsible for the emergence of a different role for *LEC1* genes in seeds plants [47]. Moreover, during the evolution of cultivated peanuts, A and B subgenomes were subjected to asymmetric homoeologous exchanges and homoeolog expression bias. Yin et al. considered that A subgenome were significantly affected by domestication, while natural selection preferred to B subgenome [48]. It was speculated that during genome evolution, to satisfy the demands for seed growth and development, the orthologous genes *AhLEC1A* and *AhLEC1B* suffered from the different selection pressure at different life stages to produce their functional divergence.

**Table 2:** Comparison of regulatory elements in *AhLEC1A* promoter and *AhLEC1B* promoter.

*cis*-element	*AhLEC1A* promoter	*AhLEC1B* promoter	Motif^a^	Putative function
Skn-1 motif	+	+	GTCAT	Cis-acting regulatory element required for endosperm expression [49]
CARGCW8GAT	+	+	CWWWWWWWWG	Motif with a longer A/T-rich core providing binding site for AGL15 which accumulates during embryo development [50]
CACTFT PPCA1	+	+	YACT	Cis-acting regulatory element required for mesophyll-specific expression [51]
-10PEHVPSBD	+	+	TATTCT	Cis-acting regulatory element involved in light responsiveness [52]
ROOT MOTIF TAPOX1	+	+	ATATT	Motif found in the promoter of rolD, which expresses strongly in roots [53]
POLLEN1 LELAT52	+	+	AGAAA	A regulatory element responsible for pollen specific activation of gene [54]
WRKY71OS	+	+	TGAC	Binding site of rice WRKY71, a transcriptional repressor of the gibberellin signaling pathway [55]
W BOX ATNPR1	+	+	TTGAC	A cluster of WRKY binding sites act as negative A regulatory element for the inducible expression of genes [56]
CPB CSPOR	+	+	TATTAG	Cis-Acting regulatory element involved in Cytokinin responsiveness [57]
DOF CORE ZM	+	+	AAAG	Core site required for binding of Dof proteins which may be associated with the plant-specific pathway for carbon metabolism [35]
E BOX BNNAPA	+	+	CANNTG	The cis-elements in the promoter regions of most genes encoding the storage protein [58]
ERE LEE4	+	+	AWTTCAAA	The ethylene responsive element mediate ethylene-induced activity of transcription [59]
SEF3 MOTIF GM	+	+	AACCCA	Binding with SEF3, one of soybean embryo factor (SEF) [37]
I BOX	+	+	GATAA	Conserved sequence upstream of light-regulated genes [41]
ARFAT	+	-	TGTCTC	Cis-Acting regulatory element involved in auxin responsiveness [60]
CAN BANAPA	+	-	CNAACAC	Core of (CA)n element in storage protein genes [32]
2S SEED PROT BANAPA	+	-	CAAACAC	Cis-Regulatory element conserved in many storage-protein gene promoters [58]
ATC-motif	+	-	AGCTATCCA	part of a conserved DNA module involved in light responsiveness [61]
ERE	+	-	ATTTCAAA	ethylene-responsive element [62]
GARE AT	+	-	TAACAAR	gibberellin-responsive element [63]
SP8BFIBSP8BIB	+	-	TACTATT	“SP8b” found in the 5’ upstream region of three different genes coding for sporamin and beta-amylase [64]
TCT motif	+	-	TCTTAC	part of a light responsive element [65]
ASF1MOTIFCAMV	-	+	TGACG	Motif involved in transcriptional activation of genes by auxin or salicylic acid, may be relevant to light regulation [66]
GCCCORE	-	+	GCCGCC	Core of GCC-box found in pathogen-responsive, ethylene-responsive and jasmonate-responsive gene [67]
TGACGTVMAMY	-	+	TGACGT	Motif required for high level expression in cotyledons of the germinated seeds[68]
5′UTR Py-rich strech	-	+	TTTCTTCTCT	Cis-acting element conferring high transcription levels [69]
CGTCA-motif	-	+	CGTCA	Cis-acting regulatory element involved in the MeJA- responsiveness [70]
GAG-motif	-	+	AGAGAGT	Part of a light responsive element [71]
GARE-motif	-	+	AAACAGA	Gibberellin-responsive element [72]
LTRE1HVBLT49	-	+	CCGAAA	“LTRE-1” (low-temperature- responsive element) in barley (H.v.) blt4.9 gene promoter [65]

Note: “+” means the element existing in the promoter, “-” means the element not existing in the promoter.

^a^W = A/T; Y = T/C; N = G/C/A/T.

In summary, we identified and characterized the promoter of *AhLEC1A*. It was found that during the process of seed development and maturation, its expression in embryo were regulated by the positive cis-elements in seed-specific mode and the negative elements restricting its expression in other organs. Moreover, *AhLEC1A* was also involved in skotomorphogenesis of peanut seeds, its expression level in the hypocotyls germinated in darkness was inhibited by some light-responsive elements. The results will be helpful for understanding the function of *AhLEC1A* in peanuts.

## Supporting information

S1 Fig Expression patterns of *AhLEC1A* in different organs. The transcription levels of *AhLEC1A* mRNA in various organs were analyzed by qRT-PCR with *AhACTIN* 7 as internal referent gene. R: Roots; St: Stems; L: Leaves; F: Flowers; S: Seeds after pegging for 30 d.
(TIF)

S1 Raw images (PDF)

## References

[1] Goldberg RB, de Paiva G, Yadegari R. Plant embryogenesis: zygote to seed. Science. 1994; 266(5185): 605–614. 10.1126/science.266.5185.605 17793455

[2] Gutierrez L, Van Wuytswinkel O, Castelain M, Bellini C. Combined networks regulating seed maturation. Trends Plant Sci. 2007; 12(7): 294–300. 10.1016/j.tplants.2007.06.003 17588801

[3] Harada JJ, Pelletier J. Genome-wide analyses of gene activity during seed development. Seed ence Research. 2012; 22(S1): S15–S22. 10.1017/s0960258511000304

[4] Braybrook SA, Harada JJ. LECs go crazy in embryo development. Trends Plant Sci. 2008; 13(12): 624–630. 10.1016/j.tplants.2008.09.008 19010711

[5] Santos-Mendoza M, Dubreucq B, Baud S, Parcy F, Caboche M, Lepiniec L. Deciphering gene regulatory networks that control seed development and maturation in Arabidopsis. Plant J. 2008; 54(4): 608–620. 10.1111/j.1365-313X.2008.03461.x 18476867

[6] Verdier J, Thompson RD. Transcriptional regulation of storage protein synthesis during dicotyledon seed filling. Plant Cell Physiol. 2008; 49(9): 1263–1271. 10.1093/pcp/pcn116 18701524

[7] Roscoe TT, Guilleminot J, Bessoule JJ, Berger F, Devic M. Complementation of Seed Maturation Phenotypes by Ectopic Expression of ABSCISIC ACID INSENSITIVE3, FUSCA3 and LEAFY COTYLEDON2 in Arabidopsis. Plant Cell Physiol. 2015; 56(6): 1215–1228. 10.1093/pcp/pcv049 25840088

[8] West M, Yee KM, Danao J, Zimmerman JL, Fischer RL, Goldberg RB, et al. LEAFY COTYLEDON1 Is an Essential Regulator of Late Embryogenesis and Cotyledon Identity in Arabidopsis. Plant Cell. 1994; 6(12): 1731–1745. 10.1105/tpc.6.12.1731 12244233 PMC160558

[9] Lotan T, Ohto M, Yee KM, West MA, Lo R, Kwong RW, et al. Arabidopsis LEAFY COTYLEDON1 is sufficient to induce embryo development in vegetative cells. Cell. 1998; 93(7): 1195–1205. 10.1016/s0092-8674(00)81463-4 9657152

[10] Harada JJ. Role of Arabidopsis LEAFY COTYLEDON genes in seed development. Journal of Plant Physiology. 2001; 158(4): 405–409. 10.1078/0176-1617-00351

[11] Calvenzani V, Testoni B, Gusmaroli G, Lorenzo M, Gnesutta N, Petroni K, et al. Interactions and CCAAT-binding of *Arabidopsis thaliana* NF-Y subunits. PLoS One. 2012; 7(8): e42902. 10.1371/journal.pone.0042902 22912760 PMC3422339

[12] Meinke DW. A Homoeotic Mutant of Arabidopsis thaliana with Leafy Cotyledons. Science. 1992; 258(5088): 1647–1650. 10.1126/science.258.5088.1647 17742538

[13] Meinke DW, Franzmann LH, Nickle TC, Yeung EC. Leafy Cotyledon Mutants of Arabidopsis. Plant Cell. 1994; 6(8): 1049–1064. 10.1105/tpc.6.8.1049 12244265 PMC160500

[14] Junker A, Mönke G, Rutten T, Keilwagen J, Seifert M, Thi TM, et al. Elongation-related functions of LEAFY COTYLEDON1 during the development of Arabidopsis thaliana. Plant J. 2012; 71(3): 427–442. 10.1111/j.1365-313X.2012.04999.x 22429691

[15] Pelletier JM, Kwong RW, Park S, Le BH, Baden R, Cagliari A, et al. LEC1 sequentially regulates the transcription of genes involved in diverse developmental processes during seed development. Proc Natl Acad Sci U S A. 2017; 114(32): E6710–e6719. 10.1073/pnas.1707957114 28739919 PMC5559047

[16] Kagaya Y, Toyoshima R, Okuda R, Usui H, Yamamoto A, Hattori T. LEAFY COTYLEDON1 controls seed storage protein genes through its regulation of FUSCA3 and ABSCISIC ACID INSENSITIVE3. Plant Cell Physiol. 2005; 46(3): 399–406. 10.1093/pcp/pci048 15695450

[17] Mu JY, Tan HL, Zheng Q, Fu FY, Liang Y, J Z. LEAFY COTYLEDON1 is a key regulator of fatty acid biosynthesis in Arabidopsis. Plant Physiol. 2008; 148(2): 1042–1054. 10.1104/pp.108.126342 18689444 PMC2556827

[18] Tan HL, Yang XH, Zhang F, Zheng X, Qu CM, Mu JY, et al. Enhanced seed oil production in canola by conditional expression of Brassica napus LEAFY COTYLEDON1 and LEC1-LIKE in developing seeds. Plant Physiol. 2011; 156(3): 1577–1588. 10.1104/pp.111.175000 21562329 PMC3135965

[19] Elahi N, Duncan RW, Stasolla C. Modification of oil and glucosinolate content in canola seeds with altered expression of Brassica napus LEAFY COTYLEDON1. Plant Physiol Biochem. 2016; 100: 52–63. 10.1016/j.plaphy.2015.12.022 26773545

[20] Tang GY, Xu PL, Ma WH, Wang F, Liu ZJ, Wan SB, et al. Seed-Specific Expression of AtLEC1 Increased Oil Content and Altered Fatty Acid Composition in Seeds of Peanut (*Arachis hypogaea* L.). Front Plant Sci. 2018; 9: 260. 10.3389/fpls.2018.00260 29559985 PMC5845668

[21] Baud S, Kelemen Z, Thévenin J, Boulard C, Blanchet S, To A, et al. Deciphering the molecular mechanisms underpinning the transcriptional control of gene expression by master transcriptional regulators in Arabidopsis seed. Plant Physiol. 2016;171(6): 1099–1112. 10.1104/pp.16.00034 27208266 PMC4902591

[22] Jo L, Pelletier JM, Hsu SW, Baden R, Goldberg RB, Harada JJ. Combinatorial interactions of the LEC1 transcription factor specify diverse developmental programs during soybean seed development. Proc Natl Acad Sci U S A. 2020; 117 (2): 1223–1232. 10.1073/pnas.1918441117 31892538 PMC6969526

[23] Brocard-Gifford IM, Lynch TJ, Finkelstein RR. Regulatory networks in seeds integrating developmental, abscisic acid, sugar, and light signaling. Plant Physiol. 2003; 131(1): 78–92. 10.1104/pp.011916 12529517 PMC166789

[24] Warpeha KM, Upadhyay S, Yeh J, Adamiak J, Hawkins SI, Lapik YR, et al. The GCR1, GPA1, PRN1, NF-Y signal chain mediates both blue light and abscisic acid responses in Arabidopsis. Plant Physiol. 2007; 143(4): 1590–1600. 10.1104/pp.106.089904 17322342 PMC1851835

[25] Siefers N, Dang KK, Kumimoto RW, Bynum WEt, Tayrose G, Holt BF 3rd. Tissue-specific expression patterns of Arabidopsis NF-Y transcription factors suggest potential for extensive combinatorial complexity. Plant Physiol. 2009; 149(2): 625–641. 10.1104/pp.108.130591 19019982 PMC2633833

[26] Huang MK, Hu YL, Liu X, Li YG, Hou XL. Arabidopsis LEAFY COTYLEDON1 Mediates Postembryonic Development via Interacting with PHYTOCHROME-INTERACTING FACTOR4. Plant Cell. 2015; 27(11): 3099–3111. 10.1105/tpc.15.00750 26566918 PMC4682307

[27] Casson SA, Lindsey K. The turnip mutant of Arabidopsis reveals that LEAFY COTYLEDON1 expression mediates the effects of auxin and sugars to promote embryonic cell identity. Plant Physiol. 2006; 142(2): 526–541. 10.1104/pp.106.080895 16935993 PMC1586040

[28] Murray MG, Thompson WF. Rapid isolation of high molecular weight plant DNA. Nucleic Acids Res. 1980; 8(19): 4321–4325. 10.1093/nar/8.19.4321 7433111 PMC324241

[29] Chen G, Shan L, Zhou LX, Tang GY, Bi YP. The Comparison of Different Methods for Isolating Total RNA from Peanuts. Chinese Agricultural ence Bulletin. 2011; 27(1): 214–218.

[30] Livak KJ, Schmittgen TD. Analysis of relative gene expression data using real-time quantitative PCR and the 2(-Delta Delta C(T)) method. Methods. 2001; 25(4):402–408. 10.1006/meth.2001.1262 11846609

[31] Jefferson RA, Kavanagh TA, Bevan MW. GUS fusions: beta-glucuronidase as a sensitive and versatile gene fusion marker in higher plants. Embo j. 1987; 6(13): 3901–3907. 3327686 10.1002/j.1460-2075.1987.tb02730.x PMC553867

[32] Ayoubi TA, Van De Ven WJ. Regulation of gene expression by alternative promoters. Faseb j. 1996; 10(4): 453–460. 8647344

[33] Bustos MM, Begum D, Kalkan FA, Battraw MJ, Hall TC. Positive and negative cis-acting DNA domains are required for spatial and temporal regulation of gene expression by a seed storage protein promoter. Embo j. 1991; 10(6): 1469–1479. 2026144 10.1002/j.1460-2075.1991.tb07667.x PMC452810

[34] Washida H, Wu CY, Suzuki A, Yamanouchi U, Akihama T, Harada K, et al. Identification of cis-regulatory elements required for endosperm expression of the rice storage protein glutelin gene GluB-1. Plant Mol Biol. 1999; 40(1): 1–12. 10.1023/a:1026459229671 10394940

[35] Yanagisawa S. Dof1 and Dof2 transcription factors are associated with expression of multiple genes involved in carbon metabolism in maize. Plant J. 2000; 21(3): 281–288. 10.1046/j.1365-313x.2000.00685.x 10758479

[36] Yanagisawa S, Schmidt RJ. Diversity and similarity among recognition sequences of Dof transcription factors. Plant J. 1999; 17(2): 209–214. 10.1046/j.1365-313x.1999.00363.x 10074718

[37] Lessard PA, Allen RD, Bernier F, Crispino JD, Fujiwara T, Beachy RN. Multiple nuclear factors interact with upstream sequences of differentially regulated beta-conglycinin genes. Plant Mol Biol. 1991; 16(3): 397–413. 10.1007/BF00023991 1893110

[38] Allen RD, Bernier F, Lessard PA, Beachy RN. Nuclear factors interact with a soybean beta-conglycinin enhancer. Plant Cell. 1989; 1(6): 623–631. 10.1105/tpc.1.6.623 2535514 PMC159797

[39] Cai M, Wei J, Li XH, Xu CG, Wang SP. A rice promoter containing both novel positive and negative cis-elements for regulation of green tissue-specific gene expression in transgenic plants. Plant Biotechnol J. 2007; 5(5): 664–674. 10.1111/j.1467-7652.2007.00271.x 17596180

[40] Müller M, Knudsen S. The nitrogen response of a barley C-hordein promoter is controlled by positive and negative regulation of the GCN4 and endosperm box. Plant J. 1993; 4(2): 343–355. 10.1046/j.1365-313x.1993.04020343.x 8220485

[41] Borello U, Ceccarelli E, Giuliano G. Constitutive, light-responsive and circadian clock-responsive factors compete for the different l box elements in plant light-regulated promoters. Plant J. 1993; 4(4): 611–619. 10.1046/j.1365-313x.1993.04040611.x 8252065

[42] Reyes JC, Muro-Pastor MI, Florencio FJ. The GATA family of transcription factors in Arabidopsis and rice. Plant Physiol. 2004; 134(4): 1718–1732. 10.1104/pp.103.037788 15084732 PMC419845

[43] Terzaghi WB, Cashmore AR. Light-Regulated Transcription. Annual Review of Plant Physiology and Plant Molecular Biology. 1995; 46(1): 445–474. 10.1146/annurev.pp.46.060195.002305

[44] Agius F, Amaya I, Botella MA, Valpuesta V. Functional analysis of homologous and heterologous promoters in strawberry fruits using transient expression. J Exp Bot. 2005; 56(409): 37–46. 10.1093/jxb/eri004 15533885

[45] Yamagata H, Yonesu K, Hirata A, Aizono Y. TGTCACA motif is a novel cis-regulatory enhancer element involved in fruit-specific expression of the cucumisin gene. J Biol Chem. 2002; 277(13): 11582–11590. 10.1074/jbc.M109946200 11782472

[46] Tang G, Xu P, Liu W, Liu Z, Shan L. Cloning and Characterization of 5’ Flanking Regulatory Sequences of AhLEC1B Gene from Arachis Hypogaea L. PLoS One. 2015; 10(10): e0139213. 10.1371/journal.pone.0139213 26426444 PMC4591277

[47] Cagliari A, Turchetto-Zolet AC, Korbes AP, Maraschin Fdos S, Margis R, Margis-Pinheiro M. New insights on the evolution of Leafy cotyledon1 (LEC1) type genes in vascular plants. Genomics. 2014; 103(5–6): 380–387. 10.1016/j.ygeno.2014.03.005 24704532

[48] Yin D, Ji C, Song Q, Zhang W, Zhang X, Zhao K, et al. Comparison of Arachis monticola with Diploid and Cultivated Tetraploid Genomes Reveals Asymmetric Subgenome Evolution and Improvement of Peanut. Adv Sci (Weinh). 2020; 7(4): 1901672. 10.1002/advs.201901672 32099754 PMC7029647

[49] Takaiwa F, Oono K, Wing D, Kato A. Sequence of three members and expression of a new major subfamily of glutelin genes from rice. Plant Mol Biol. 1991; 17(4): 875–885. 10.1007/BF00037068 1680490

[50] Tang W, Perry SE. Binding site selection for the plant MADS domain protein AGL15: an in vitro and in vivo study. J Biol Chem. 2003; 278(30): 28154–28159. 10.1074/jbc.M212976200 12743119

[51] Gowik U, Burscheidt J, Akyildiz M, Schlue U, Koczor M, Streubel M, et al. cis-Regulatory elements for mesophyll-specific gene expression in the C4 plant Flaveria trinervia, the promoter of the C4 phosphoenolpyruvate carboxylase gene. Plant Cell. 2004; 16(5): 1077–1090. 10.1105/tpc.019729 15100398 PMC423201

[52] Thum KE, Kim M, Morishige DT, Eibl C, Koop HU, Mullet JE. Analysis of barley chloroplast psbD light-responsive promoter elements in transplastomic tobacco. Plant Mol Biol. 2001; 47(3): 353–366. 10.1023/a:1011616400264 11587507

[53] Elmayan T, Tepfer M. Evaluation in tobacco of the organ specificity and strength of the rolD promoter, domain A of the 35S promoter and the 35S2 promoter. Transgenic Res. 1995; 4(6): 388–396. 10.1007/BF01973757 7581519

[54] Filichkin SA, Leonard JM, Monteros A, Liu PP, Nonogaki H. A novel endo-beta-mannanase gene in tomato LeMAN5 is associated with anther and pollen development. Plant Physiol. 2004; 134(3): 1080–1087. 10.1104/pp.103.035998 14976239 PMC389932

[55] Zhang ZL, Xie Z, Zou X, Casaretto J, Ho TH, Shen QJ. A rice WRKY gene encodes a transcriptional repressor of the gibberellin signaling pathway in aleurone cells. Plant Physiol. 2004; 134(4): 1500–1513. 10.1104/pp.103.034967 15047897 PMC419826

[56] Chen C, Chen Z. Potentiation of developmentally regulated plant defense response by AtWRKY18, a pathogen-induced Arabidopsis transcription factor. Plant Physiol. 2002; 129(2): 706–716. 10.1104/pp.001057 12068113 PMC161695

[57] Fusada N, Masuda T, Kuroda H, Shimada H, Ohta H, Takamiya K. Identification of a novel cis-element exhibiting cytokinin-dependent protein binding in vitro in the 5’-region of NADPH-protochlorophyllide oxidoreductase gene in cucumber. Plant Mol Biol. 2005; 59(4): 631–645. 10.1007/s11103-005-0579-x 16244912

[58] Stålberg K, Ellerstöm M, Ezcurra I, Ablov S, Rask L. Disruption of an overlapping E-box/ABRE motif abolished high transcription of the napA storage-protein promoter in transgenic Brassica napus seeds. Planta. 1996; 199(4): 515–519. 10.1007/BF00195181 8818291

[59] Tapia G, Verdugo I, Yañez M, Ahumada I, Theoduloz C, Cordero C, et al. Involvement of ethylene in stress-induced expression of the TLC1.1 retrotransposon from Lycopersicon chilense Dun. Plant Physiol. 2005; 138(4): 2075–2086. 10.1104/pp.105.059766 16040666 PMC1183396

[60] Hagen G, Guilfoyle T. Auxin-responsive gene expression: genes, promoters and regulatory factors. Plant Mol Biol. 2002; 49(3–4): 373–385. 10.1023/A:1015207114117 12036261

[61] Welsch R, Medina J, Giuliano G, Beyer P, Von Lintig J. Structural and functional characterization of the phytoene synthase promoter from Arabidopsis thaliana. Planta. 2003; 216(3): 523–534. 10.1007/s00425-002-0885-3 12520345

[62] Rawat R, Xu ZF, Yao KM, Chye ML. Identification of cis-elements for ethylene and circadian regulation of the Solanum melongena gene encoding cysteine proteinase. Plant Mol Biol. 2005; 57(5): 629–643. 10.1007/s11103-005-0954-7 15988560

[63] Ogawa M, Hanada A, Yamauchi Y, Kuwahara A, Kamiya Y, Yamaguchi S. Gibberellin biosynthesis and response during Arabidopsis seed germination. Plant Cell. 2003; 15(7): 1591–1604. 10.1105/tpc.011650 12837949 PMC165403

[64] Ishiguro S, Nakamura K. Characterization of a cDNA encoding a novel DNA-binding protein, SPF1, that recognizes SP8 sequences in the 5’ upstream regions of genes coding for sporamin and beta-amylase from sweet potato. Mol Gen Genet. 1994; 244(6): 563–571. 10.1007/BF00282746 7969025

[65] Luo QL, Li YG, Gu HQ, Zhao L, Gu XP, Li WB. The promoter of soybean photoreceptor GmPLP1 gene enhances gene expression under plant growth regulator and light stresses. Plant Cell, Tissue and Organ Culture (PCTOC). 2013; 114(1): 109–119. 10.1007/s11240-013-0310-6

[66] Redman J, Whitcraft J, Johnson C, Arias J. Abiotic and biotic stress differentially stimulate as-1 element activity in Arabidopsis. Plant Cell Reports. 2002; 21(2): 180–185. 10.1007/s00299-002-0472-x

[67] Chakravarthy S, Tuori RP, D’Ascenzo MD, Fobert PR, Despres C, Martin GB. The tomato transcription factor Pti4 regulates defense-related gene expression via GCC box and non-GCC box cis elements. Plant Cell. 2003; 15(12): 3033–3050. 10.1105/tpc.017574 14630974 PMC282854

[68] Yamauchi D. A TGACGT motif in the 5’-upstream region of alpha-amylase gene from Vigna mungo is a cis-element for expression in cotyledons of germinated seeds. Plant Cell Physiol. 2001; 42(6): 635–641. 10.1093/pcp/pce079 11427683

[69] Ye J, Li WF, Ai G, Li CX, Liu GZ, Chen WF, et al. Genome-wide association analysis identifies a natural variation in basic helix-loop-helix transcription factor regulating ascorbate biosynthesis via D-mannose/L-galactose pathway in tomato. PLoS Genet. 2019; 15(5): e1008149. 10.1371/journal.pgen.1008149 31067226 PMC6527244

[70] Wang Y, Liu GJ, Yan XF, Wei ZG, Xu ZR. MeJA-inducible expression of the heterologous JAZ2 promoter from Arabidopsis in Populus trichocarpa protoplasts. J PLANT DIS PROTECT. 2011.

[71] Jin B, Sheng ZL, Ishfaq M, Chen JQ, Yang HL. Cloning and functional analysis of the promoter of a stress-inducible gene (Zmap) in maize. PLoS One. 2019; 14(2): e0211941. 10.1371/journal.pone.0211941 30735543 PMC6368321

[72] Sutoh K, Yamauchi D. Two cis-acting elements necessary and sufficient for gibberellin-upregulated proteinase expression in rice seeds. Plant J. 2003; 34(5): 635–645. 10.1046/j.1365-313x.2003.01753.x 12787245

