# Supplementary figures and images for: Cloning and functional characterization of seed-specific LEC1A promoter from peanut (Arachis hypogaea L.)
Source: PLoS One. 2021 Mar 22;16(3):e0242949. doi: 10.1371/journal.pone.0242949 (PMC7984638; doi:10.1371/journal.pone.0242949)

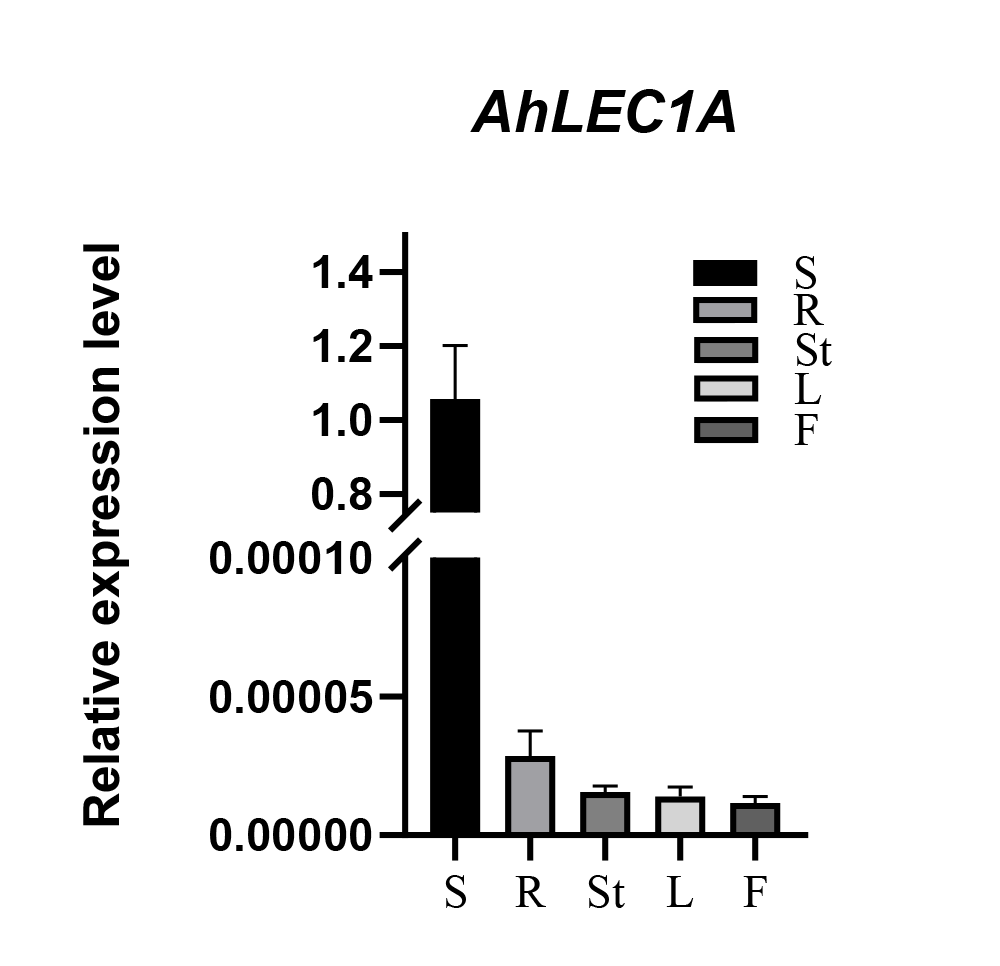

Supplement: S1 Fig — The transcription levels of AhLEC1A mRNA in various organs were analyzed by qRT-PCR with AhACTIN 7 as internal referent gene. R: Roots; St: Stems; L: Leaves; F: Flowers; S: Seeds after pegging for 30 d. (TIF) [file pone.0242949.s001.tif]
