# Supplementary material for: Cloning and functional characterization of seed-specific LEC1A promoter from peanut (Arachis hypogaea L.)
Source: PLoS One. 2021 Mar 22;16(3):e0242949. doi: 10.1371/journal.pone.0242949 (PMC7984638; doi:10.1371/journal.pone.0242949)
Supplement: S1 Raw images — (PDF) [file pone.0242949.s002.pdf]

1.

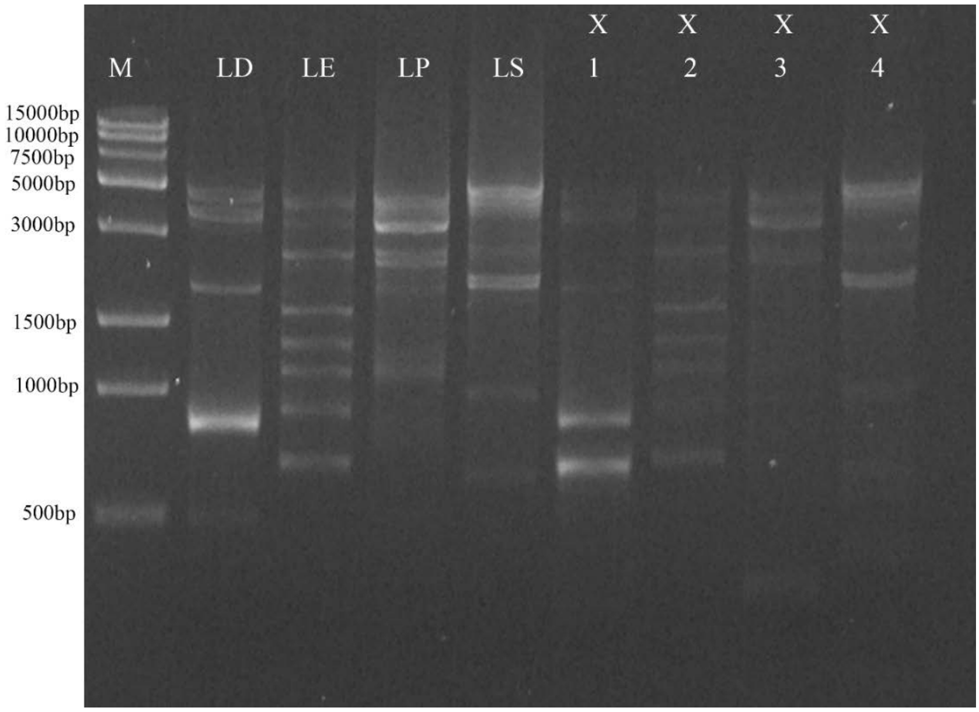

The gel image is the original one of the Figure 1 in our manuscript. The left 5 lanes labeling M, LD, LE, LP, LS were cropped as the Figure 1, but not including the right 4 lanes labeling 1-4.

2.

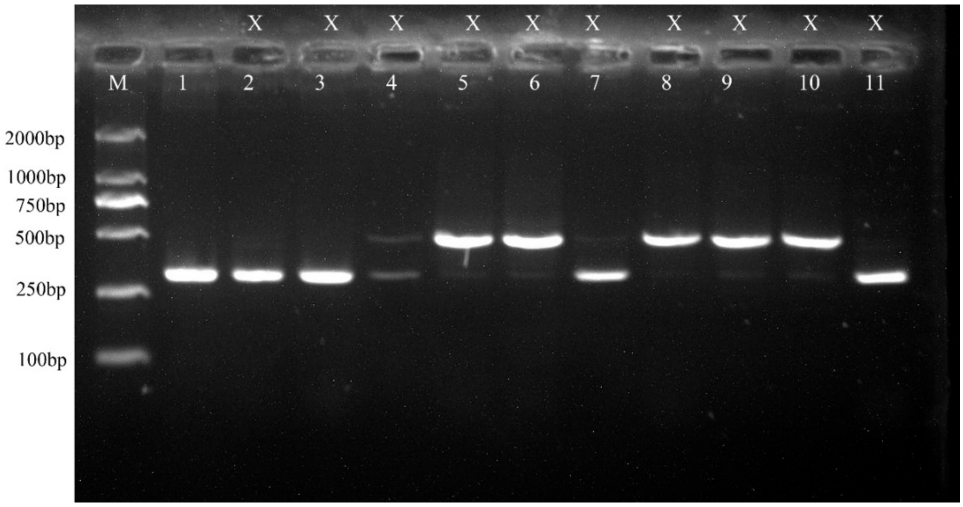

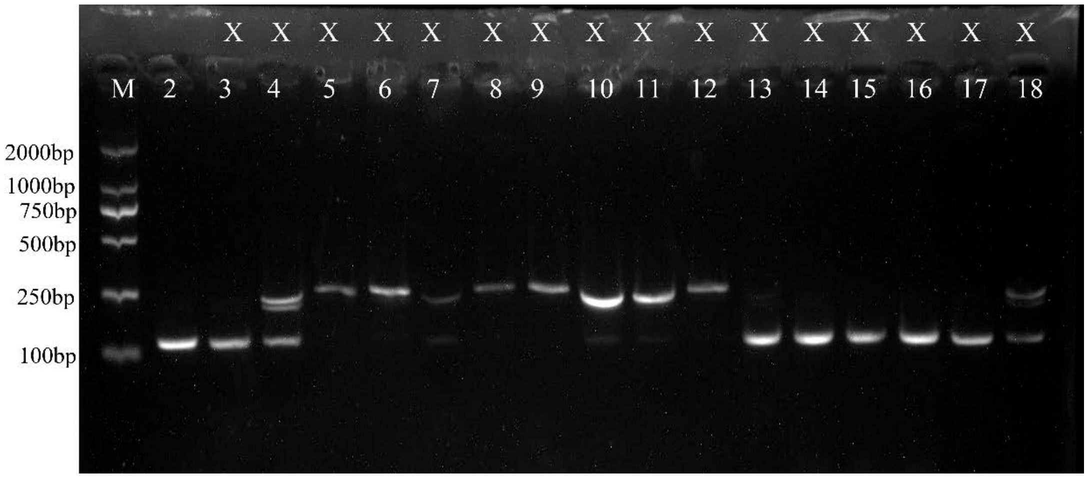

The Figure 2 in our manuscript was derived from the above two original figures. The left one of Figure 2 was cropped from the left two lanes M and 1 of the first figure, and the right one was cropped from the left two lanes M and 2 of the second figure.
